# Safety, efficacy and prognosis of anticoagulant therapy for portal vein thrombosis in cirrhosis: a retrospective cohort study

**DOI:** 10.1186/s12959-023-00454-x

**Published:** 2023-01-30

**Authors:** Zhiqi Zhang, Ying Zhao, Dandan Li, Mingxing Guo, Hongyu Li, Ranjia Liu, Xiangli Cui

**Affiliations:** 1 Department of Pharmacy, Beijing Friendship Hospital, Capital Medical University, Beijing, China; 2 College of Pharmacy, Capital Medical University, No. 10, West Toutiao, Beijing, China; 3 Liver Transplantation Center, National Clinical Research Center for Digestive Diseases, Beijing Friendship Hospital, Capital Medical University, Beijing, China

**Keywords:** Anticoagulant, Portal vein thrombosis, Liver cirrhosis

## Abstract

**Background:**

The role of anticoagulants in the treatment of cirrhotic PVT remains controversial. This study aimed to analyze the safety and efficacy of anticoagulant therapy in patients with cirrhotic portal vein thrombosis (PVT) and its impact on prognosis.

**Methods:**

A retrospective cohort study was conducted for PVT patients with liver cirrhosis in our hospital. The primary outcome of the study was the PVT recanalization rate. Other outcomes included bleeding rate, liver function, and mortality. Cox and Logistic regression were used to explore the risk factors of outcomes.

**Results:**

This study included 77 patients that 27 patients in the anticoagulant group and 50 in the non-anticoagulant group. Anticoagulant therapy was associated with higher rate of PVT recanalization (44.4% vs 20.0%, log-rank *P* = 0.016) and lower rate of PVT progression (7.4% vs 30.0%, log-rank *P* = 0.026), and without increasing the rate of total bleeding (14.8% vs 24%, *P* = 0.343), major bleeding (3.7% vs 6%, *P* = 0.665) and variceal bleeding (3.7% vs 16%, *P* = 0.109). The safety and efficacy of different anticoagulants were similar. The Child-Pugh grade of the anticoagulant therapy group was better than that of the non-anticoagulant therapy group (*P* = 0.030). There was no significant difference in the 2-year survival rate of the two groups.

**Conclusion:**

Anticoagulants could increase the PVT recanalization rate and reduce the PVT progression rate without increasing the rate of bleeding. Anticoagulants may be beneficial to improving the liver function of patients with cirrhotic PVT. There was no significant difference in the safety and efficacy of different anticoagulants in the treatment of cirrhotic PVT.

## Background

Portal vein thrombosis (PVT) is one of the severe/major complications of liver cirrhosis, the incidence is reported to be 5% ~ 20% [1, 2]. The pathogenesis of PVT included increased vascular resistance and a low flow rate of the portal vein. Liver cirrhosis generally led to portal hypertensive, and esophageal-gastric variceal bleeding [3]. The occurrence and progression of PVT will further increase portal vein resistance and aggravate portal hypertension [4]. Besides, for patients taking liver transplantation, PVT complicates the operation and is associated with higher post-transplant mortality [5, 6]. Anticoagulant therapy for cirrhotic PVT remains controversial. Several studies [7–9] have reported spontaneous recanalization of cirrhotic PVT due to the coagulation factors and fibrinogen levels decreased in patients with liver cirrhosis [10]. Besides, the prognostic value of PVT on cirrhosis outcome is still an unsolved issue [11]. Some other studies [12, 13] supported anticoagulant therapy because it was suggested to increase the portal vein recanalization rate without increasing the bleeding rate. A previous meta-analysis [14] suggested that anticoagulant therapy can increase the recanalization rate of PVT without increasing the bleeding risk. But there was a limited number of studies included, and the heterogeneity among studies was high. Thus, it should be further investigated the benefit and risks associated with anticoagulant treatment for patients with cirrhotic PVT. In this study, we aimed to analyze the safety and efficacy of anticoagulants for patients with cirrhotic PVT through a cohort study in a tertiary hospital.

## Methods

### Study cohort

Patients with cirrhotic PVT who were admitted to the Liver disease Center of our hospital from January 2015 to December 2021 were retrospectively evaluated. The inclusion criteria were as follows: (1) age ≥ 18 years; (2) liver cirrhosis was diagnosed according to the criteria of the Japanese Society of Gastroenterology (JSGE) [15]; and (3) PVT was diagnosed by abdominal Doppler ultrasound, magnetic resonance imaging (MRI) and computed tomography (CT). The exclusion criteria were: (1) malignant related PVT; (2) isolate splenic or mesenteric venous thrombosis; (3) those receiving non-anticoagulant treatment such as transjugular intrahepatic portosystemic shunt (TIPS), antithrombotic, thrombolysis, or thrombectomy during liver transplantation; (4) platelet count < 10 × 10^9^/L; (5) creatinine clearance ≤30 mL/min; (6) primary thrombophilia; (7) Budd-Chiari syndrome; (8) pregnancy or breast-feeding women; (9) severe cardiopulmonary diseases; (10) cases without imaging follow-up information.

Eligible patients were divided into two groups according to the administration of anticoagulants or not. The anticoagulant group included patients that use anticoagulants for PVT treatment, including warfarin, rivaroxaban, dabigatran, or low molecular weight heparin (LMWH) without limitations on dosing. The non-anticoagulant group included patients that did not use any anticoagulants for PVT treatment. Patients with a history of variceal bleeding received endoscopic esophageal varix ligation (EVL) and carvedilol to reduce portal pressure. Ethical approval for this retrospective study was obtained from Beijing Friendship Hospital, Capital Medical University.

Review of medical records of all enrolled patients and collection of baseline information from the records. The information collected included gender, age, etiology of liver cirrhosis, type and duration of anticoagulants, presence or absence of hepatocellular carcinoma (HCC), history of liver transplantation, indicators of liver function, location of PVT, and the degree of PVT occlusion. The indicators of liver function included all variables to evaluate the Child-Pugh and Model for End-Stage Liver Disease (MELD) scores. Location and degree of PVT occlusion were evaluated according to the report of abdominal MRI or CT.

### Follow-up and clinical end-points

Patients were followed until death, liver transplantation, or the end of the study (February 2022). In case of loss to follow-up, patients were followed until the last record within our health system [7, 16]. MRI or CT was performed every 6 months to check the recanalization of PVT. The primary outcome was the rate of PVT recanalization including both complete and partial recanalization. Complete recanalization referred to the complete disappearance of the thrombus and partial recanalization referred to more than 50% reduction of the thrombus. The secondary outcomes were bleeding, the progress of liver function, PVT progression, and mortality. Bleedings included major bleeding that meets the criteria of the International Society on Thrombosis and Haemostasis (ISTH) [17], variceal bleeding, and any bleeding. PVT progression was defined as more than 25% extension of the thrombus or distribution of thrombus increased.

### Statistical analysis

The sample size and the power of the test were calculated by PASS (version 15, NCSS, LLC. USA). The testing level α was set as 0.05, and the expected testing power 1-β was set as 0.9. The rate of PVT recanalization refers to a previous meta-analysis [14] that the recanalization rate of the treatment group was 66.7% and the rate of the control group was 26%. By pre-reviewing the cases in our hospital, the number of patients who received no anticoagulant therapy for cirrhotic PVT was roughly double that of those who received anticoagulant therapy. Thus the group allocation was set as N2 / N1 = 2. At least 21 patients needed to be included in the anticoagulant group and the non-anticoagulant group needed to include at least 42 patients.

Data analyses were carried out using SPSS (version 26, IBM Corp, USA). Qualitative variables were expressed as frequency and percentage and were compared using the χ^2^ test or Fischer’s exact test. Continuous variables that accorded with normal distribution were expressed as mean ± standard deviation, and analyzed using the independent sample t-test; continuous variables that did not accord with the normal distribution were expressed by median (inter-quartile range) and analyzed by the non-parametric test. Logistic regression and Cox regression analysis were used to explore the risk factors of outcomes. *P*-value < 0.05 of two-sided was considered statistically significant. Kaplan-Meier survival curve was used to analyze the probability of PVT recanalization and progression over time in anticoagulant and non-anticoagulant groups.

## Results

### Baseline characteristics of included studies

**Fig. 1:**
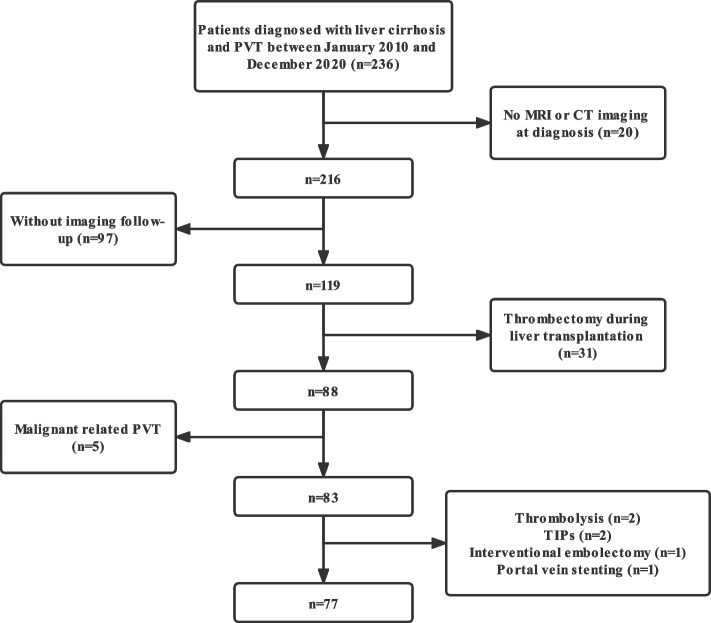
Patients screening Flow chart

236 patients with cirrhotic PVT were initially identified. After screening, 77 patients met the the inclusion and exclusion criteria (Fig. 1), including 27 in the anticoagulant group and 50 in the non-anticoagulant group. Anticoagulants used included warfarin with the INR target level of 1.5–2.5 (*n* = 6), nadroparin 4100 U qd (*n* = 2), heparin 12,500 U qd (*n* = 1), rivaroxaban 20 mg qd (*n* = 3), rivaroxaban 10 mg qd (*n* = 14), edoxaban 30 mg qd (*n* = 1).

Anticoagulant therapy was started within 3 months of estimated PVT onset in 24 (69%) of 27 patients, whereas in 3 patients, anticoagulant therapy was initiated after 8, 21, and 36 months. The median follow-up time was 26 months (IQR 13–44 months) and did not differ significantly between anticoagulant and non-anticoagulant groups (18 vs 28.5 months, *P* = 0.071). The median time of imaging follow-up was 10 months (IQR 5.5–22.5 months) and did not differ significantly between the two groups (10 vs 10.5 months, *P* = 0.712). A total of 24 of 27 patients (88.9%) who received anticoagulants eventually discontinued therapy. Patients discontinuing anticoagulant therapy because of PVT recanalization (*n* = 10), bleeding complication (*n* = 4), no response after treatment for more than 6 months (*n* = 3), decreased treatment adherence (*n* = 3), worsening clinical status (*n* = 1) and not clearly documented (*n* = 3). The median duration of anticoagulant therapy was 6 (IQR 2–11) months. The baseline characteristics of the included patients are shown in Table 1.

**Table 1:** Baseline characteristics of included patients

	Non-anticoagulant group (***n*** = 50)	Anticoagulant group (***n*** = 27)	***P***
**Gender**			0.215
Male	26 (52.0%)	18 (66.7%)	
Female	24 (48.0%)	9 (33.3%)	
**Age (years, mean ± SD)**	59.0 ± 13.0	60.4 ± 12.3	0.641
**Etiology**			0.693
HBV	14 (28.0%)	10 (37.0%)	
PBC	13 (26.0%)	3 (11.1%)	
Alcohol	11 (22.0%)	7 (25.9%)	
NASH	2 (4.0%)	2 (7.4%)	
Drug	2 (4.0%)	1 (3.7%)	
Other	8 (16.0%)	4 (14.8%)	
**HCC**	2 (4.0%)	2 (7.4%)	0.917
**History of splenectomy**	5 (10.0%)	11 (40.7%)	**0.002**
**Child-Pugh score**			0.102
A	16 (34.0%)	15 (55.6%)	
B	24 (51.1%)	11 (40.7%)	
C	7 (14.9%)	1 (3.7%)	
**MELD (mean ± SD)**	6.4 ± 5.5	5.2 ± 4.0	0.316
**Laboratory tests**			
ALB (g/L)	32.9 ± 4.8	34.9 ± 5.0	0.094
Median bilirubin (umol/L, IQR)	23.6 (18.4–31.0)	19.8 (14.4–29.9)	0.318
Median ALT (U/L, IQR)	18.5 (12.0–27.5)	18.0 (14.0–30.0)	0.906
Median AST (g/L, IQR)	32.7 (22.5–41.6)	27.3 (21.6–35.6)	0.175
Median creatinine (IQR)	69.0 (60.0–93.8)	71.6 (57.8–93.6)	0.498
Median PT (s, IQR)	14.1 (13.2–15.7)	14.3 (12.7–14.7)	0.328
INR	1.3 ± 0.2	1.2 ± 0.1	0.222
Median Platelet count (×10^9^/L, IQR)	74.0 (52.0–102.0)	97.0 (69.0–195.0)	**0.041**
Hemoglobin (g/L)	98.8 ± 22.3	110.1 ± 22.2	**0.040**
D-dimer (ug/mL, IQR)	2.5 (1.3–4.3)	3.2 (1.7–6.2)	0.484
**Degree of PV occlusion**			0.753
Occlusive	0 (0.0%)	1 (3.7%)	
Non-occlusive	50 (100.0%)	26 (96.3%)	
**Location of PVT**			
Main PV	42 (84.0%)	22 (81.5%)	1.000
Intrahepatic branch of PV	21 (42.0%)	15 (55.6%)	0.255
splenic vein	13 (26.0%)	8 (30.8%)	0.659
Mesenteric vein	16 (32.0%)	12 (44.4%)	0.279

*SD* Standard Deviation; *IQR* interquartile range; *HBV* hepatitis B virus; *PBC* primary biliary cirrhosis; *NASH* non-alcoholic steatohepatitis; *ALD* autoimmune liver disease; *HCC* hepatocellular carcinoma; *MELD* model for end stage liver disease; *ALB* albumin; *ALT* alanine aminotransferase; *AST* aspartate aminotransferase; *PT* prothrombin time; *APTT* active partial thromboplastin time; *INR* international standard ratio; *PVT* portal vein thrombosis; *PV* portal vein

### Rate of PVT recanalization

**Fig. 2:**
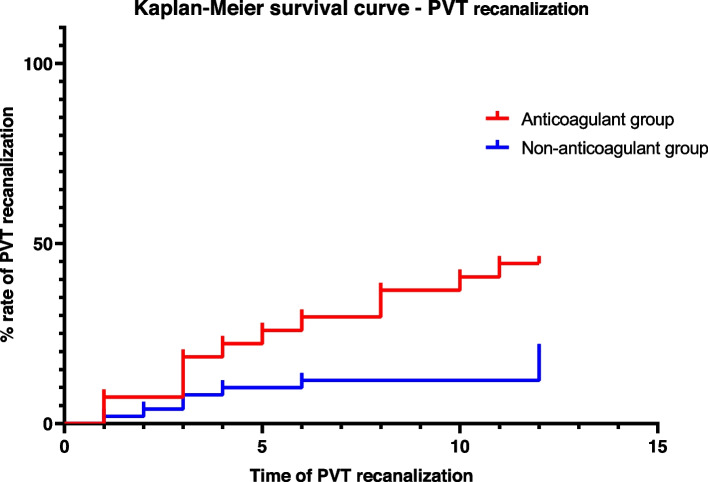
Kaplan-Meier survival curve of PVT recanalization

A Kaplan-Meier curve depicting the probability of PVT recanalization over time among patients who did and did not receive anticoagulants is depicted in Fig. 2. A total of 12/27 (44.44%) patients who received anticoagulants experienced PVT recanalization compared with 10/50 (20%) of patients who did not receive anticoagulants (log-rank *P* = 0.016). One patient in the anticoagulant group had complete PVT recanalization, and 11 patients had partial PVT recanalization. In the non-anticoagulant group, complete recanalization of PVT occurred in 6 patients, and partial PVT recanalization for 4 patients. Multiple cox regression analysis adjusted by the history of splenectomy, platelet count, and hemoglobin suggested that the application of anticoagulants was associated with a significantly higher rate of PVT recanalization (HR 2.672, 95% CI 1.151–6.203, *P* = 0.022). There was no significant difference in PVT recanalization rate among different anticoagulants including warfarin, heparin, and DOACs (Table 2).

**Table 2:** Cox regression analysis of PVT recanalization

Anticoagulants	HR	95% CI	log-rank ***P***
**DOACs vs warfarin**	4.045	0.517–37.668	0.143
**heparin vs warfarin**	1.826	0.114–29.273	0.666
**DOACs vs heparin**	2.150	0.275–16.835	0.448

**Fig. 3:**
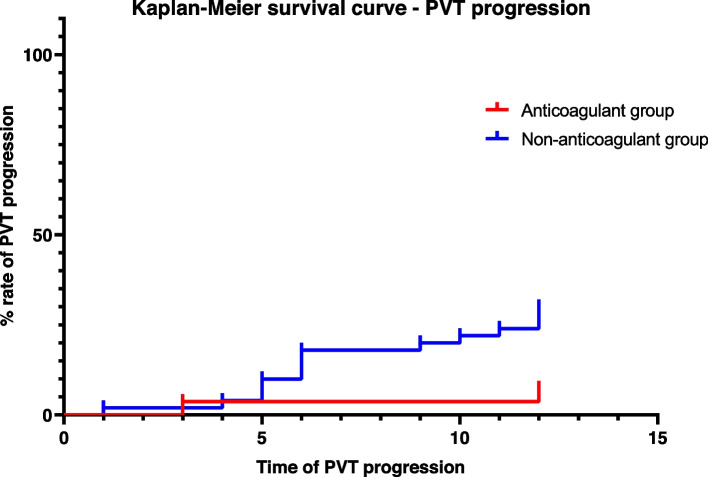
Kaplan-Meier survival curve of PVT progression

PVT progression occurred among 13/27 (7.4%) of those who received anticoagulants compared with 15/50 (30%) of those who did not (log-rank *P* = 0.026) (Fig. 3). The multiple cox regression analysis suggested that the application of anticoagulants was associated with a significantly lower rate of PVT progression (HR 0.221, 95% CI 0.051–0.969, *P* = 0.017).

### Safety of anticoagulant therapy

Five bleeding episodes among 4 patients (14.8%) occurred during follow-up after anticoagulant therapy. One patient had variceal bleeding and hematochezia, and other patients had upper gastrointestinal bleeding, abnormal uterine bleeding, and major variceal bleeding, respectively. All patients in the anticoagulant group stopped anticoagulant treatment after bleeding complications. One patient developed thrombocytopenia when receiving rivaroxaban 10 mg qd, and continued treatment by reducing the frequency of administration to 10 mg qod. A total of 17 bleeding events occurred in 12 patients in the non-anticoagulant group during follow-up. One patient had four variceal bleeding and two patients had once variceal bleeding and once melena. The other 5 patients developed once variceal bleeding and 4 patients had once melena. Among the 8 patients with variceal bleeding, 3 were major bleeding. There were no significant differences in the incidence of total bleeding (14.8% vs 24%, *P* = 0.343), major bleeding (3.7% vs 6%, *P* = 0.665) and variceal bleeding (3.7% vs 16%, *P* = 0.109) between anticoagulant group and non-anticoagulant group. There was no significant difference in the incidence of total bleeding, variceal bleeding, and major bleeding among warfarin, heparin, and DOACs (Table 3).

**Table 3:** Comparison of bleeding events with different anticoagulants

Anticoagulants	Total bleeding	*P*	Major bleeding	*P*	Variceal bleeding	*P*
DOACs vs warfarin	2/18 vs 1/6	1.000	1/18 vs 0/6	1.000	1/18 vs 0/6	1.000
heparin vs warfarin	1/3 vs 1/6	1.000	0/3 vs 0/6	–	0/3 vs 0/6	–
DOACs vs heparin	2/18 vs 1/3	0.386	1/18 vs 0/3	1.000	1/18 vs 0/3	1.000

The significant predictor of variceal bleeding was the history of variceal bleeding. Among 34 patients with a history of variceal bleeding, 8 patients had variceal bleeding during follow-up (23.5%); Of the 40 patients without a history of variceal bleeding, 1 developed variceal bleeding during follow-up (2.5%), and the difference was statistically significant (OR = 12.0 95% CI 1.416–101.714, *P* = 0.023).

### Prognosis

At the end of the follow-up, there were 12 patients of Child-Pugh A, 12 of Child-Pugh B, and 1 of Child-Pugh C in the anticoagulant group. And in the non-anticoagulant group, there were 12 patients of Child-Pugh A, 24 of Child-Pugh B, and 11 of Child-Pugh C. The classification of liver function has a significant difference between the anticoagulant group and the non-anticoagulant group (*P* = 0.030) after follow-up. But there was no significant difference in the 2-year survival rate between the two groups (*P* = 1.000). One patient in the anticoagulant group died of liver failure 8 months after the diagnosis of PVT. In the non-anticoagulant group, one patient died of abdominal infection 9 months after the diagnosis, and one patient died of liver failure 24 months after the diagnosis. None of these deaths were related to bleeding complications.

## Discussion

Due to the uncertain effect of anticoagulant therapy on the prognosis of patients with cirrhotic PVT, the usage of anticoagulants for cirrhotic PVT remains controversial. Besides, anticoagulants for cirrhotic PVT have a limited suitable population recommended by the guidelines [18], and the optimal anticoagulants remain undetermined. This is due to insufficient clinical data on the safety and efficacy of anticoagulants for cirrhotic PVT. We analyzed patients in our institution to understand the current situation of anticoagulants for cirrhotic PVT and provide more experience in anticoagulant therapy.

Spontaneous recanalization of PVT was observed in patients of the non-anticoagulant group, but the recanalization rate was higher in patients receiving anticoagulant therapy, which means anticoagulants appear effective for the treatment of cirrhotic PVT. The recanalization rate of the anticoagulant group in our study was lower than that found in a previous meta-analysis (66.2%) [14], which might be related to the dosage of anticoagulant regimens. In our study, rivaroxaban was the most commonly used anticoagulant, but only 3 patients received the therapeutic dose of 20 mg qd, while most used 10 mg qd. And the patients taking warfarin had an INR goal of 1.5–2.5. The inadequate anticoagulant dosage may be responsible for the low recanalization rate of PVT. There was no significant difference in PVT recanalization rate and bleeding rate between patients taking warfarin and rivaroxaban in our study. However, an RCT [16] suggested that rivaroxaban 10 mg q12h had a higher rate of PVT recanalization than warfarin (complete response 70% vs 20%, *P* < 0.001). Thus, we need to clarify the relationship between the dosage and the efficacy and safety of different anticoagulants first, and then compare the differences among the anticoagulants.

Clinicians prefer to use low-dose anticoagulants, which may be due to the worry of bleeding complications. However, our study concluded that anticoagulants did not increase the rate of total bleeding, major bleeding, and variceal bleeding. This may indicate that anticoagulant therapy is safe for patients with cirrhotic PVT. ACG clinical guidelines [18] also indicated that anticoagulant therapy was not associated with an increased risk of variceal bleeding in patients with cirrhotic PVT, and the presence of gastroesophageal varices was not a contraindication to anticoagulant therapy. A previous meta-analysis [19] even concluded that anticoagulation can reduce the rate of variceal bleeding(OR 0.232, 95% CI 0.06–0.94, *P* = 0.04). The mechanism may be related to anticoagulants that can reduce pressure in oesophageal varices [20].

There is also uncertainty about the effect of anticoagulant therapy on the prognosis of patients with cirrhotic PVT. The presence of PVT increased the complexity of liver transplantation surgery and increased the risk of early mortality after liver transplantation [6]. However, it is not clear whether anticoagulant therapy affects patient outcomes in patients who have not undergone liver transplantation. A multicenter, long-term follow-up of cirrhotic PVT showed no significant difference in Kaplan-Meier survival curves of overall survival after 5 years of follow-up between the anticoagulant and non-anticoagulant groups (83% vs 70%, log-rank *P* = 0.1362). After 2 years of follow-up, there was also no difference in mortality between the two groups was observed in our study. A recent meta-analysis [21] showed that the survival rate of the anticoagulant group was higher than that of the non-anticoagulant group (OR 1.11, 95% CI 1.03–1.21, *P* = 0.010), but it was not clear whether patients underwent liver transplantation during the follow-up period. Therefore, more studies are needed to evaluate the efficacy and safety of anticoagulant therapy in patients with cirrhotic PVT who are not awaiting liver transplantation to guide the optimal anticoagulant therapy.

In terms of the prognosis of liver function, we observed that there was no significant difference in the baseline Child-Pugh score between the anticoagulant group and the non-anticoagulant group, but there was a significant difference in liver function score between the two groups at the end of follow-up. Child-Pugh grade C patients increased and grade A patients decreased in the non-anticoagulant group. An RCT study found [9] that the Child-Pugh score of patients after anticoagulant therapy was significantly improved compared with before (7 vs 6, *P* = 0.007). So anticoagulant therapy may have a beneficial effect on the liver function of patients with cirrhotic PVT. Besides, it is considered that the safety of DOACs in patients with Child-Pugh grade C needs to be evaluated. A Child-Pugh grade C patient included in our study had good safety and effectiveness after taking rivaroxaban for 8 months, but larger sample studies with larger sample sizes are still needed to collect more data on the safety of DOACs in those patients.

There were several limitations of our study. Firstly, this single-center study had a limited number of patients, which may lead to biased results. But it fulfilled the statistical demand. Secondly, anticoagulants therapy could increase levels of PT(s) and INR (part of Child-Pugh score and MELD score respectively), which may result in underestimation of liver function. But this is an inevitable confounding factor in the evaluation of liver function. Thirdly, we have followed patients for a median of 26 months, longer follow-ups are needed to observe the effect of anticoagulation on the mortality of patients with cirrhotic PVT.

In conclusion, anticoagulant therapy could increase the rate of PVT recanalization without increasing the rate of bleeding in patients with liver cirrhosis and could reduce the rate of variceal bleeding. Compared with the non-anticoagulant group, anticoagulant therapy may be beneficial to the liver function of patients with cirrhotic PVT. There was no significant difference in the safety and efficacy of different anticoagulants in the treatment of cirrhotic PVT. Further studies are needed to optimize the use of anticoagulants in patients with cirrhotic PVT.

## Data Availability

Not applicable.
